# Ecological constraint, rather than opportunity, promotes adaptive radiation in three‐spined stickleback (*Gasterosteus aculeatus*) on North Uist

**DOI:** 10.1002/ece3.9716

**Published:** 2023-01-10

**Authors:** Mahmuda Begum, Victoria Nolan, Andrew D. C. MacColl

**Affiliations:** ^1^ School of Life Sciences University of Nottingham Nottingham UK; ^2^ Zoology Section, Biological Research Division Bangladesh Council of Scientific & Industrial Research (BCSIR) Dhaka Bangladesh

**Keywords:** morphometric study, North Uist, South Uist, stickleback

## Abstract

The context and cause of adaptive radiations have been widely described and explored but why rapid evolutionary diversification does not occur in related evolutionary lineages has yet to be understood. The standard answer is that evolutionary diversification is provoked by ecological opportunity and that some lineages do not encounter the opportunity. Three‐spined sticklebacks on the Scottish island of North Uist show enormous diversification, which seems to be associated with the diversity of aquatic habitats. Sticklebacks on the neighboring island of South Uist have not been reported to show the same level of evolutionary diversity, despite levels of environmental variation that we might expect to be similar to North Uist. In this study, we compared patterns of morphological and environmental diversity on North and South Uist. Ancestral anadromous sticklebacks from both islands exhibited similar morphology including size and bony “armor.” Resident sticklebacks showed significant variation in armor traits in relation to pH of water. However, North Uist sticklebacks exhibited greater diversity of morphological traits than South Uist and this was associated with greater diversity in pH of the waters of lochs on North Uist. Highly acidic and highly alkaline freshwater habitats are missing, or uncommon, on South Uist. Thus, pH appears to act as a causal factor driving the evolutionary diversification of stickleback in local adaptation in North and South Uist. This is consistent with diversification being more associated with ecological constraint than ecological opportunity.

## Graphical abstract

Evolutionary diversification is often thought to arise from “ecological opportunity:” A reduction in competition for resources following the invasion of a new habitat. Here, we show that diversification may be provoked by ecological constraint: Organisms invading habitats, in this case, acid freshwater, may be free from competition but have poor resource availability.

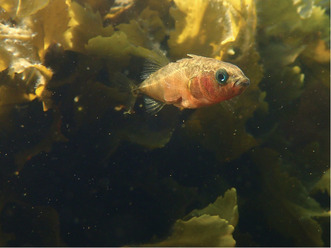

## 1 INTRODUCTION

Studies of adaptive radiation have tended to concentrate, for obvious reasons, on lineages and locations in which it is known to have occurred. The circumstances associated with a failure to radiate are less well explored, yet there is much that might be learned from them about the conditions that favor divergence. Differences in ecological conditions among local populations are generally assumed to be the reason for adaptive divergence leading to adaptive radiation, reproductive isolation, and speciation (MacColl, 2011; Schluter, 1996; Schluter, 2000). In particular, according to the ecological theory of adaptive radiation (Schluter, 2000), “ecological opportunity,” the diversity of available resources not used by other taxa, is central to explaining why adaptive radiations occur. Lineages often diversify when they colonize habitats where there is little competition for constraining resources (Schluter, 2000; Simpson, 1953). The concentration on biotic interactions, especially competition for food, that has followed from the idea of ecological opportunity, has meant that the role of abiotic environmental variation has been less well explored. At least some adaptive radiations involve diversification into harsh, unproductive environments such as high mountains or deep cold water (Barrier et al., 1999; Ingram & Kai, 2014; Mangel et al., 2007; Robichaux et al., 1990). There may be fewer competitors in such environments, but resources are nevertheless scarce, such that species that occupy them grow slowly and at low density. These circumstances do not chime with a standard perception of ecological opportunity: while there may be little competition, the evolution of divergent taxa appears to be associated with fewer resources, suggesting ecological constraint rather than opportunity. In the latter case (opportunity), we might expect to see rapid growth of both populations and individuals relative to their ancestors, at least in the early stages of colonization of new environments. In the former (constraint), we would expect to see slower growth. Here, we scrutinize diversification of three‐spined sticklebacks (*Gasterosteus aculeatus*) in lochs on two neighboring Scottish islands, for clues about the causes of adaptive radiation.

The three‐spined stickleback (*Gasterosteus aculeatus*, hereafter “stickleback”) has served as a model organism for the study of adaptive radiation due in part to parallel diversifications of freshwater populations from marine ancestors that have occurred in the last 10,000 years (Bell & Foster, 1994; Jones et al., 2012; Magalhaes et al., 2021; Schluter, 2000). Freshwater populations have evolved conspicuous differences in morphology, physiology, and behavior (Bell & Foster, 1994; Ostlund‐Nilsson et al., 2006). The divergence of morphological traits through major changes in the bony armor that have repeatedly evolved in various locations are common components of adaptive radiation of stickleback (Chan et al., 2010; Colosimo et al., 2004). Ancestral marine anadromous sticklebacks are heavily armored with a continuous row of 30–36 bony lateral plates running from head to tail on each side (known as a complete morph), and have dorsal spines, a well‐developed pelvic girdle and further spines attached to the girdle (Barrett et al., 2008; Colosimo et al., 2005). On the other hand, derived freshwater (live in freshwater year round) and saltwater‐resident (inhabit coastal saltwater all year round without migration to the open sea) sticklebacks (Dean et al., 2019; McKinnon et al., 2004; Taylor & McPhail, 2000) exhibit substantial reduction in the total plate number with either a discontinuous row of 9–28 plates (partial morph) or with 0–9 lateral plates at the anterior end (low morph), and reductions in the size of spines and girdle (Colosimo et al., 2004; Colosimo et al., 2005). In addition, other phenotypic traits such as body size and shape of freshwater and saltwater‐resident sticklebacks show morphological transformation from the ancestral anadromous form (Bell et al., 1993; Moodie & Reimchen, 1976; Ravinet et al., 2015; Schluter, 1993; Schluter et al., 2004; Shapiro et al., 2004).

To date, several abiotic and biotic environmental factors have been considered as causes for these major phenotypic changes including variations in salinity, temperature, nutrient and calcium availability, stream gradient, competition, predators, and parasites (Barrett et al., 2009; Bergstrom, 2002; Giles, 1983; Marchinko, 2009; Miller et al., 2019; Myhre & Klepaker, 2009; Wark & Peichel, 2010). For example, predation‐driven selection has been reported to influence the evolution of bony armor structures such as the lateral plates and spines within freshwater stickleback populations (Marchinko, 2009). Moreover, other factors, such as reduced nutrients or salinity, and calcium ion deficiency have also been reported to have an association with the loss of lateral plates in freshwater populations (Barrett et al., 2009; Giles, 1983; Myhre & Klepaker, 2009; Rennison et al., 2019).

The neighboring islands of North and South Uist, in the Scottish Western Isles, appear to have much in common from the perspective of aquatic habitats and species. Both have large numbers of small‐ to medium‐sized shallow lochs that occur over a variety of surface geology, from peat and bedrock on their eastern sides to shell‐rich machair sand on their western sides. Although fish, including three‐spined stickleback, populations have been surveyed on both islands (Campbell, 1985; Campbell & Williamson, 1979), detailed studies of phenotypic variation in the sticklebacks have only taken place on North Uist (Giles, 1983; MacColl et al., 2013; Magalhaes et al., 2016; Magalhaes et al., 2021), apparently because of a lack of such variation on South Uist (Campbell, 1985).

North Uist comprises a mosaic of interconnected freshwater and brackish water lochs and lagoons which are known to have exceptional variation in their water chemistry, with high pH (~8) in the western machair lochs and low pH (~6) in the east. These are associated with variations in the concentrations of alkaline metals (sodium, potassium, magnesium, calcium, etc.) (Waterston et al., 1979). This variation has been shown to correlate with the evolution of high diversity of stickleback populations across the island (Giles, 1983; Haenel et al., 2019; MacColl et al., 2013; Magalhaes et al., 2016) and offers a unique opportunity to study the variation in phenotypes in relation to adaptation under different environmental conditions.

In contrast, environmental conditions and phenotypic variation have been poorly explored in South Uist, and there have been no reports of unusual morphological variation in sticklebacks. While different in topography (it has higher hills and less low‐lying ground), it appears to have similar variation in aquatic environments, with both saltwater and freshwater (<1‰ salt) lochs, and both western, machair lochs and eastern lochs on peat or bedrock. This begs the question why, on two such closely neighboring islands, where ancestral variation is likely to be shared, and environmental variation is similar, there appears to have been little or no diversification of stickleback on one, while the other exhibits some of the most dramatic variation known in the species. Here, we make a direct comparison of the morphological and environmental variation in the two islands, and relate the former to the latter, to understand what factors may promote or inhibit adaptive radiation at local scales. We hypothesized that morphological traits of three‐spined sticklebacks would show variation between South Uist and North Uist due to differences in the environmental diversity of the lochs.

## 2 MATERIALS AND METHODS

### 2.1 Study area

**FIGURE 1:**
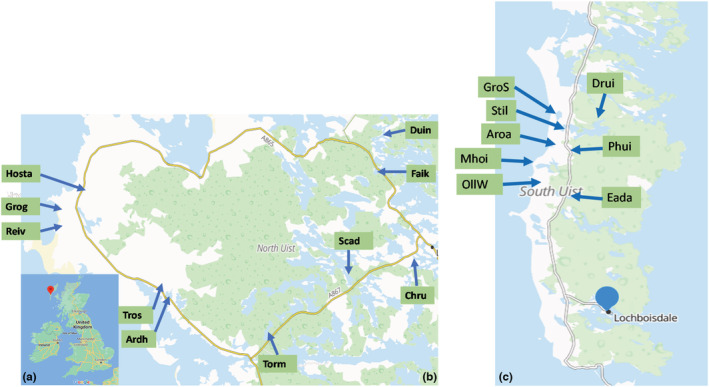
Sites for the collection of samples from 10 lochs in North Uist and 8 lochs in South Uist, Scotland [(a) Map of United Kingdom (b) Loch position in North Uist (c) Loch position in South Uist with road map (yellow), agricultural land (white), and moorland (green)]

Two neighboring islands with apparently similar aquatic environments were selected to make a comparative study of morphological variation in three‐spined sticklebacks. North Uist (57°31′12″N; 7°27′42″W) is in the center of the Western Isles of Scotland and is approximately 303 km^2^ in total area (Figure 1b). It comprises a mosaic of peat bogs, heathland, and low hills. South Uist (57°13′54″N; 7°02′38″W) is the second largest island of the Outer Hebrides (Figure 1c). It is around 320 km^2^ in total area and differs greatly between its west (low‐lying) and east (hilly) sides.

### 2.2 Sample collection

Stickleback samples were collected from 10 lochs on North Uist and 8 lochs on South Uist between the dates of 6th and 16th May 2019 (Figure 1). Locations on both islands were selected to maximize the likely variation in surface geology (sand vs. peat or bedrock), and hence water chemistry, using Google Earth. Sticklebacks were caught in unbaited Gee's Minnow traps (Gee traps, Dynamic Aqua, Vancouver, Canada) set overnight (approximately 16 h.) at all sites. The geolocation of each sample site (GPSmap 60CSx, Garmin, UK) and water quality parameters including absolute conductivity, salinity, and pH (multi‐parameter probe – Multi340/set, WTW, Germany) were recorded from all sites (Tables S1 and S2). Lochs were grouped based on the pH of water: high pH >7.5, neutral pH = >7.0–7.4, and low pH <7.0 (Tables S1 and S2).

Across all 10 sampling sites of North Uist, 135 sticklebacks were haphazardly selected (Figure 1b). From South Uist, 128 fish were collected in the same way from 11 sites (3 sites from loch Aroa) in eight lochs (Figure 1c). Of the total 263 sticklebacks, 45 were anadromous fish (live in the sea but migrate to fresh/brackish water to breed) sampled from both islands to estimate the ancestral state of stickleback in the Uist lochs. Fish were euthanized and preserved in 70% ethanol for morphometric study.

### 2.3 Body armor and spine data collection for morphometric analysis

To collect data on external bony skeletal structures (armor), ethanol‐preserved samples were stained with Alizarin Red solution following a standard staining method (Peichel et al., 2001). Samples were stored in 40% isopropyl alcohol (propan‐2‐ol). After confirming the appearance of bony parts, a digital photograph of the right side of every fish was captured using a digital SLR camera (Nikon D5200) with 60 mm macro lens, digital ring flash, and a tripod (to set a fixed distance). All photographs included graph paper as a scale (cm) and the measurements of standard length (from the tip of the snout to the end of caudal peduncle); first and second dorsal spine, pelvic spine (from insertion point to the tip), pelvis height, and pelvis length were recorded using tpsDig2 version 2.31 (Rohlf, 2010) (Figure S1 in Appendix S1). The total number of lateral plates was also counted from the right side of the stained photograph.

### 2.4 Data analysis

Data were collated in Excel (Microsoft) and statistical analyses were conducted using R, version 3.6.3 (R Core Team, 2020), and Genstat version 22.1.

### 2.5 Variation in morphological traits between North Uist and South Uist

In order to avoid confounding variation in armor with overall variation in size, all measured armor traits (except number of lateral plates) were size standardized by calculating the residuals of a regression of each trait against standard length to obtain their allometric relationship with body size. The resident (including freshwater) (hereafter “resident”) and anadromous fish were analyzed separately to quantify variation in morphological data (lateral plate count [hereafter “plate count”], standard length, residuals of first dorsal spine length [first dorsal spine], second dorsal spine length [second dorsal spine], pelvic spine length [pelvic spine], length of pelvis [pelvis length], and height of pelvis [pelvis height]). We used principal component analysis (PCA) to describe efficiently the overall variation in morphological variables between islands and pH bands (high, neutral, and low). PCA was performed using the singular value decomposition method to explore the axis of greatest variation in the measured armor data. All variables were scaled and centered for PCA and grouped for visualization purposes according to three variables: location (North Uist and South Uist), pH (high pH, neutral pH, and low pH), and salinity (freshwater and saltwater). Subsequent analyses were carried out on the size‐standardized residuals for individual morphological traits since we believe that these are clearer and easier to interpret.

We compared both trait means and overall variation in traits (coefficients of variation, CV) between the islands. The first reveals whether there are overall differences in the magnitude of traits that might suggest average differences in environments, or explain differences in patterns of variation. The second tells us about the patterns of variation in traits (rather than trait means) across populations. Generalized linear mixed models (GLMMs, with Gaussian distributions and identity link functions) were used to test for mean differences in each size standardized morphological trait between the two islands (North Uist and South Uist). “Loch” was fitted as a random term. Modified signed‐likelihood ratio tests (MSLRT) (Krishnamoorthy & Lee, 2014) were used to test for differences in coefficients of variation (CV) between North Uist and South Uist for population (loch) means of each morphological trait. Pearson's correlations were used to test for relationships between measured armor traits. To compare the mean fish length of the anadromous and resident sticklebacks, a Wilcoxon rank‐sum t‐test was performed for all samples of North Uist and South Uist.

### 2.6 Environmental factors affecting the variation in morphological traits in North Uist and South Uist

To quantify associations between morphological traits of resident stickleback and different environmental factors of the loch water in North Uist and South Uist, GLMs were fitted with a Gaussian distribution and identity link function. First, the average of PC1 and PC2 from the PCA of armor traits of each population were fitted as response variables, with location (North or South Uist), pH value, salinity, and conductivity as predictor variables. Then, population mean and size‐standardized morphological traits (standard length, first dorsal spine length, second dorsal spine length, pelvic spine length, length of pelvis, height of pelvis, and plate count) were fitted as response variables, with the same explanatory variables. Stepwise regression with a combination of forward and backward selection based on likelihood‐ratio tests was conducted for all GLM models. The best‐fitting model was then selected based on Akaike's information criterion (AIC). All models were checked for goodness of fit using quantile–quantile (Q‐Q) plots of the residuals, and the significance of each component was tested using ANOVA tests. A Scheffe post hoc test (Scheffe, 1951) for multiple comparisons of means with unequal sample sizes was performed to test the differences in the size of measured armor traits between the unusual values in Tros and three other low pH lochs on North Uist.

## 3 RESULTS

### 3.1 Variation in morphological traits between North Uist and South Uist

**FIGURE 2:**
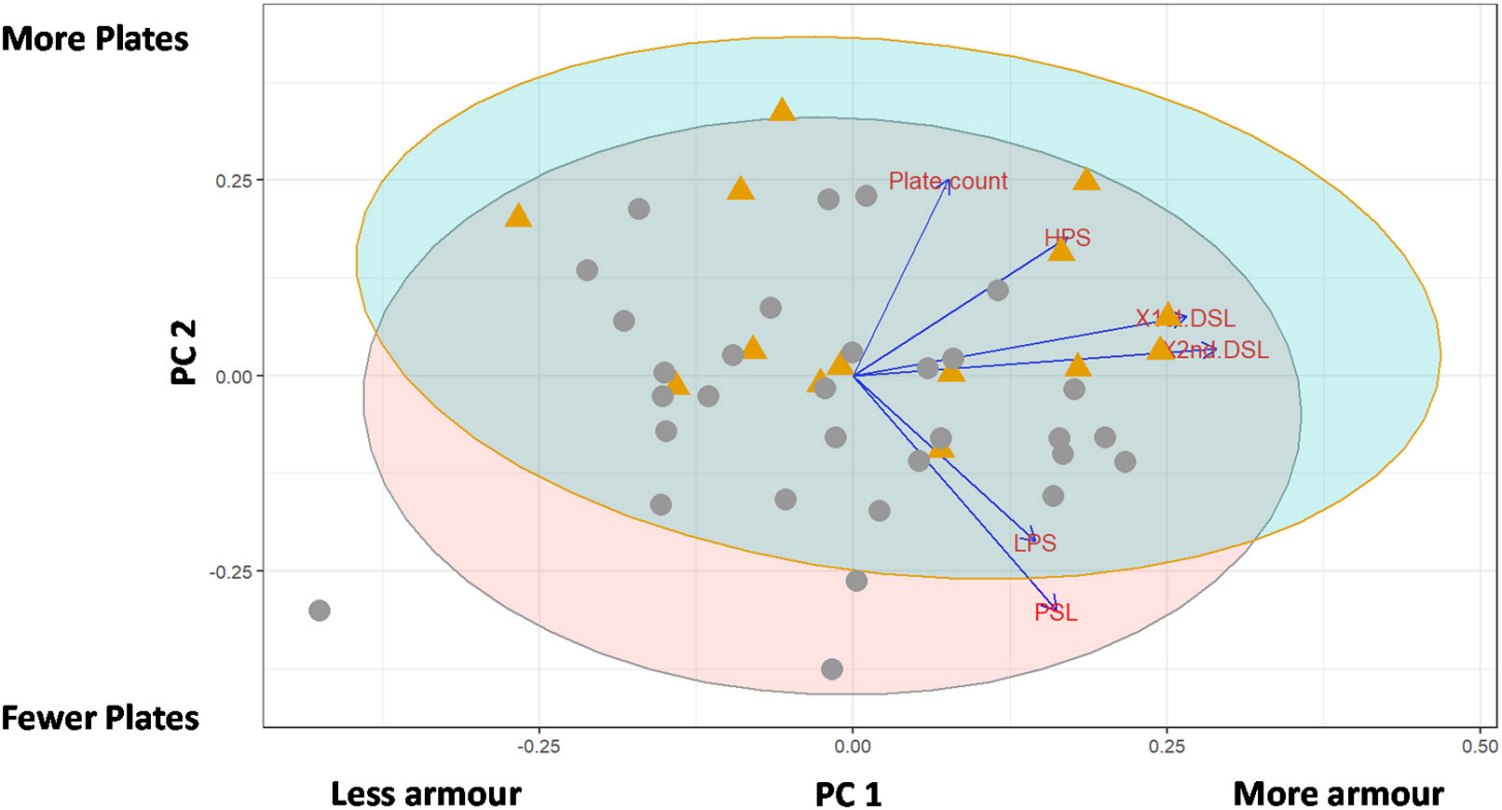
The first two PCs of body armor traits for anadromous sticklebacks collected from North (ash, circle) and South (orange, triangle) Uist. Traits were plate count and residual values (from body length) of first dorsal spine length (X1st.DSL), second dorsal spine length (X2nd.DSL), pelvic spine length (PSL), length of pelvis (LPS), and height of pelvis (HPS). PC1 (35.2%) describes overall variation in armor, with positive loadings of all variables. PC2 (22.75%) mainly not only describes variation in plate count (positive loading) but also reflects negative loadings for LPS and PSL. Ellipses represent 95% confidence levels within each dataset

The first two PCs from the PCA of the armor traits for the anadromous stickleback from both North and South Uist accounted for 58% of the total variation. All measured armor traits had positive loadings for PC1 and PC2 except pelvic spine length and length of pelvis (Figure 2). Overall, anadromous fish from North and South Uist were morphologically similar, with one exception. The mean height of the pelvis of South Uist anadromous fish was significantly greater than North Uist (Table 1).

**TABLE 1:** Comparison of means and coefficients of variation (CVs) in morphological traits between North and South Uist

Morphological traits	North and South Uist									
	Anadromous					Resident				
	Mean			CV		Mean			CV	
	Wald F	*df*	*p*	MSLRT	*p*	Wald F	*df*	*p*	MSLRT	*p*
Std. length	1.03	1, 4.7	.77	0.46	.50	2.59	1, 16.2	.13	0.27	.60
First DSL	0.09	1, 2.1	.79	1.25	.26	0.42	1, 16.3	.53	10.29	**.001**
Second DSL	0.02	1, 2.7	.90	3.79	.05	0.19	1, 16.2	.67	11.26	**<.001**
PSL	2.97	1, 43	.09	0.20	.66	0.34	1, 16.2	.57	8.78	**.003**
LPS	0.18	1, 2.7	.71	−0.03	1.00	0.41	1, 16.1	.53	5.05	**.02**
HPS	16.6	1, 43	**<.001**	0.19	.19	0.66	1, 16.1	.48	10.64	**.001**
Plate count	0.68	1, 4.4	.45	0.11	.17	0.15	1, 16.4	.70	2.86	.09

*Note*: Generalized linear mixed models (GLMM) were used to test differences in mean trait values, with “population” as a random effect. Modified signed‐likelihood ratio tests (MSLRT) were used to test differences in CVs of trait means between the islands. Morphological traits were standard length, first dorsal spine length (DSL), second dorsal spine length (DSL), pelvic spine length (PSL), length of pelvis (LPS), height of pelvis (HPS), and plate count] of stickleback sample fish (217 residents and 45 anadromous). Armor traits, except plate counts, were standardized for overall size variation, by taking residuals from regressions of each trait against standard length. Significant *p* values are in bold.

**FIGURE 3:**
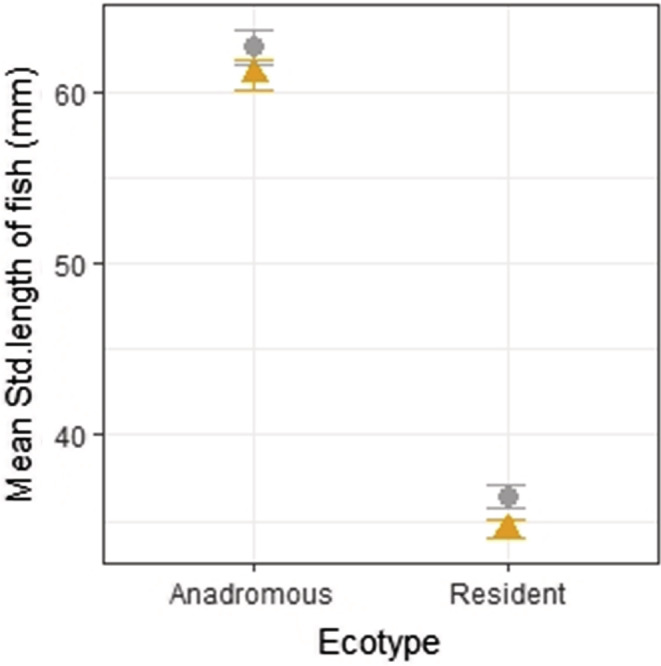
Mean standard length (±SE) of anadromous and resident fish shows little overall variation in size between North (ash, circle) and South Uist (orange, triangle) sticklebacks. Resident sticklebacks of North and South Uist were significantly smaller than the anadromous fish

Resident populations of sticklebacks from North and South Uist were significantly smaller (smaller mean standard length) than the anadromous populations (W = 9742, *p* < .001; Figure 3). The mean standard length of the North Uist resident stickleback (mean ± SE. 36.44 ± 0.64 mm) was significantly greater than South Uist fish (34.55 ± 0.53 mm) (one‐way ANOVA: *F* = 5.21, *df* = 1, 216, *p* = .02; Figure 3), although the difference in length is small.

**FIGURE 4:**
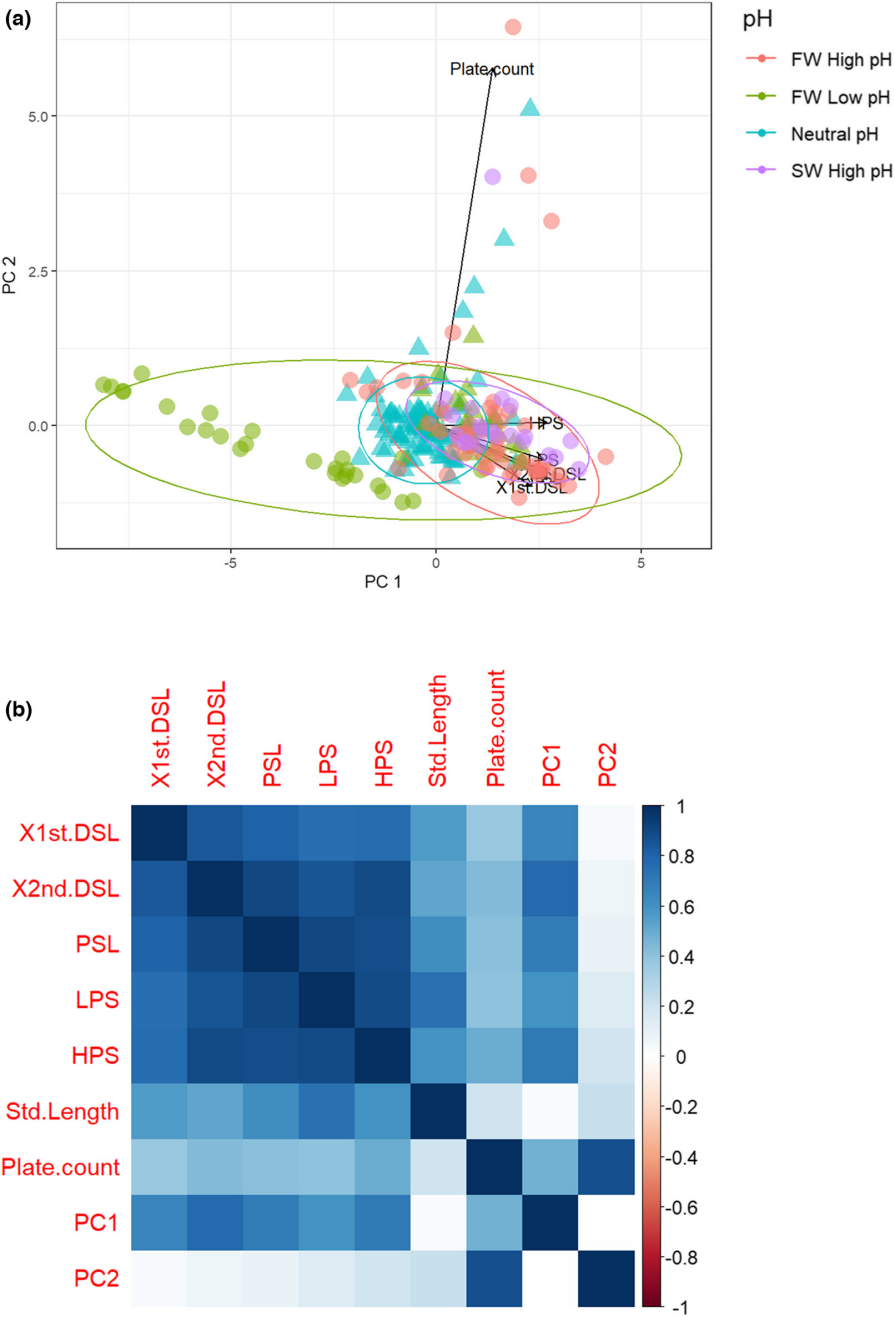
(a) The first two principal components (PCs) of six body armor traits and standard length for resident sticklebacks of North Uist (circle) and South Uist (triangle). PC1 (71.3%) describes overall variation in spine length and pelvic size, while PC2 (13.8%) describes variation in plate number. Each dot represents a single fish in one of four pH groups: Freshwater high pH (red), freshwater low pH (green), neutral pH (turquoise), and saltwater high pH (purple). Ellipses describe 95% confidence intervals for each pH group. (b) Correlogram showing correlations between all measured armor traits including plate count, PC1, PC2, and standard length of North and South Uist resident fish. Blue color denotes positive correlations where shade of the square box indicates the strength of the correlation. Measured traits were as follows: first dorsal spine length (X1st.DSL), second dorsal spine length (X2nd.DSL), pelvic spine length (PSL), length of pelvis (LPS), and height of pelvis (HPS)

The first two principal components of a PCA of armor traits for resident fish collected from North and South Uist explained approximately 85% of the variance among individuals (Figure 4a). PC1 was strongly correlated with all measures of armor traits so high values of PC1 are associated with more armor. Plate count was highly correlated with PC2 and had a stronger influence on the variation in North Uist fish than South Uist. All measured armor traits (except plate count) were strongly correlated with each other (Pearson's correlation (*r*) > .5, Figure 4b). For resident fish, despite no significant differences in trait means, there was significantly more variation (CV) between populations in all traits, except standard length and plate count in North Uist than South Uist (Table 1).

### 3.2 Factors affecting the variation in morphological traits in North Uist and South Uist

There was wide variation in many of the environmental factors including salinity and pH across the 8 lochs of South Uist and 10 lochs of North Uist: loch pH ranged from low (6.5, acidic) to high (~9, alkaline) pH of water (one‐way ANOVA: *F* = 10.28, *df* = 1, 216, *p* = .001) in the freshwater lochs of North Uist compared to mostly neutral (~7) lochs of South Uist (Tables S1 and S2). Across all lochs, there was little relationship between pH, salinity and conductivity (Figure S1).

There were significant associations between the first two PCs of armor traits and the pH of the 18 populations of resident fish collected from North and South Uist (Table 2), but not with the other environmental variables. There was striking morphological variation in fish found in acidic freshwater lochs relative to other lochs (Figure 4).

**TABLE 2:** Summary of significant relationships between morphological and environmental variation in resident stickleback across North and South Uist lochs

Response variable	Predictor variable	Family	Link function	Population	*F*	*df*	*p*
Avg. PC1	pH value	Gaussian	Identity	18	4.88	1, 17	**.042**
Avg. PC2	pH value	Gaussian	Identity	18	5.37	1, 17	**.034**
Avg. standard length	pH value	Gaussian	Identity	18	4.85	1, 17	**.044**
Avg. plate count	pH value	Gaussian	Identity	18	11.3	1, 17	**.003**
Avg. first dorsal spine	pH value	Gaussian	Identity	18	4.46	1, 17	.050.
Avg. second dorsal spine	pH value	Gaussian	Identity	18	4.83	1, 17	**.043**
Avg. pelvic spine	pH value	Gaussian	Identity	18	7.42	1, 17	**.015**
Avg. length of pelvis	pH value	Gaussian	Identity	18	11.3	1, 17	**.004**
Avg. height of pelvis	pH value	Gaussian	Identity	18	10.4	1, 17	**.005**

*Note*: Results show minimum adequate models from GLMs, with loch average values of armor PCs or individual traits fitted as response variables, and pH, salinity, and conductivity as explanatory variables. Significance at *p* < .05 is denoted in bold.

**FIGURE 5:**
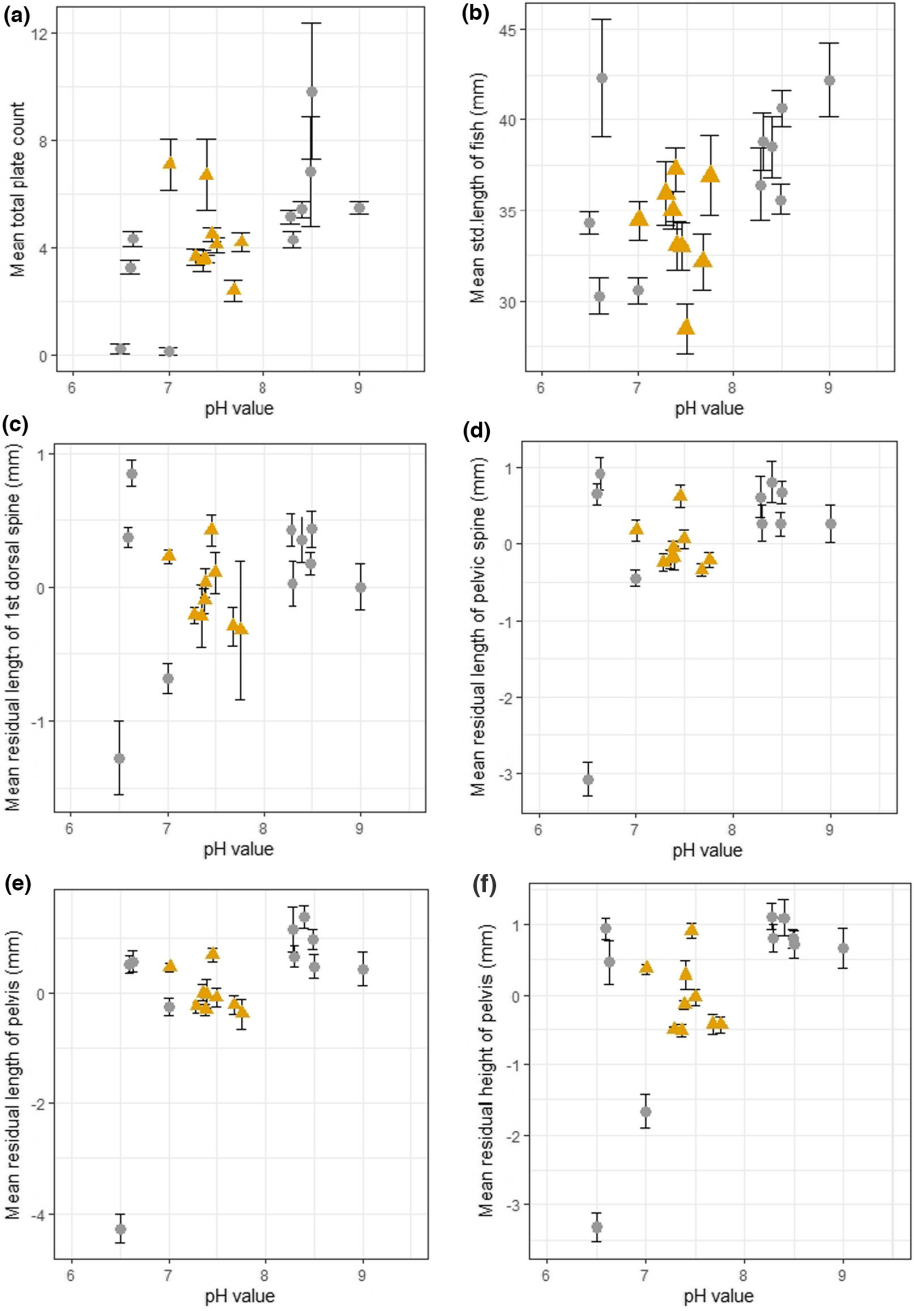
(a–f) mean (±SE) plate count (a) and standard length (b) and mean residual size (c–f) of five armor traits in 18 freshwater stickleback populations on North (circle) and South Uist (triangle)

Consistent with the patterns apparent in the PCA plot, all morphological traits of the resident fish showed significant associations with pH in both North and South Uist (Table 2), but not with the other environmental variables. The overall mean plate count across both North and South Uist was highest in the high pH populations (6.58 ± 1.41) and lowest in freshwater low pH lochs of North Uist (1.86 ± 0.27) (Table 2; Figure 5a). The mean standard length of resident fish was highest in freshwater high pH populations (37.02 ± 0.97 mm) and lowest in freshwater low pH (33.49 ± 0.92 mm) (Table 2; Figure 5b). Among the 18 populations of resident fish of both islands, the mean length of all measured armor variables increased with increasing pH (Figure 5c–f). One exception to the overall trend was in Tros (pH 6.63), where armor measurements were relatively large. Post hoc tests (Scheffe test) revealed that all measured armor traits in Tros were significantly longer than in the three other freshwater low pH populations of North Uist (*p* < .001).

## 4 DISCUSSION

Studies of adaptive radiation have tended to concentrate on what we can learn from adaptive radiations themselves, rather than their absence. In this study, we have compared lacustrine populations of three‐spined sticklebacks from isolated habitats on two neighboring Scottish islands. North Uist has been known for decades to support a striking example of early adaptive radiation in this species (Campbell, 1985; Magalhaes et al., 2021), apparently linked to variation in the abiotic and biotic environments that are indexed by variation in pH (Giles, 1983; Haenel et al., 2019; MacColl et al., 2013; Magalhaes et al., 2016). In contrast, variation in sticklebacks on South Uist has hardly been considered, despite apparently similar variation in aquatic environments. We have shown that in fact, South Uist lacks the striking morphological variation in North Uist, and this lack of evolutionary diversification is well explained by a lack of variation in the pH of water bodies.

Anadromous sticklebacks that are likely to provide a good approximation for the ancestors of Uist freshwater sticklebacks showed similar variation in armor traits and size on North and South Uist, consistent with close common ancestry in the marine environment, supporting the idea that differences between the two islands arise because of differences in freshwater. In contrast, resident fish collected from North and South Uist showed variation in all measured armor traits within and among populations which indicates that there is adaptive radiation of phenotypic characters among populations as a result of colonization and adaptation to the freshwater environment (Bell & Foster, 1994; Magalhaes et al., 2021). There was significantly more variation in armor traits in resident fish of North Uist than South Uist, which demonstrates differences in evolutionary diversification of closely related lineages.

The pH of the lochs showed strong associations with the armor traits of resident fish collected from North and South Uist consistent with several previous studies of stickleback adaptive evolution in freshwater environments (Bell & Foster, 1994; Giles, 1983; MacColl et al., 2013; Magalhaes et al., 2016; Spence et al., 2012). In the present study, the armor traits of resident sticklebacks showed substantial variation in dorsal spines, pelvic spines, and pelvis length in the freshwater populations of both islands. However, the North Uist sticklebacks exhibited much more morphological variation than the sticklebacks of South Uist and this variation was directly associated with greater diversity in pH of the loch water on North Uist. This suggests that the differing extent of morphological diversification between North and South Uist is a direct consequence of differences in some aspect or aspects of the environment that are related to pH.

The greater morphological variation on North Uist is accounted for by the presence there of low pH lochs, especially Scad and Torm that appear to be missing from South Uist. The sticklebacks in these lochs are certainly unusual in the wider, global spectrum of stickleback variation (Haenel et al., 2019; Magalhaes et al., 2021). The sticklebacks in these oligo‐ to dystrophic lochs are unusually small (MacColl et al., 2013), likely for nutritional reasons, and in Scad and Torm, completely lack some elements of armor. It is difficult to square this with the idea that these habitats provide an ecological opportunity, in the normal understanding of that term, which implies an abundance of resources. It is true that there is little competition in the acid lochs on North Uist (MacColl et al., 2013), but the habitat appears to drive unusual phenotypic variation because of nutritional constraints, rather than opportunities.

It is not immediately obvious why there should be less environmental variation in South, than in North, Uist. At first sight, the surface geology of both islands, the root cause of the pH variation (Waterston et al., 1979), is rather similar, with acidic, peaty lochs in the east and alkaline, machair lochs in the west. On North Uist, the acidic lochs, where the most extreme phenotypes have evolved, are relatively large. Adjacent, smaller lochs with similar pH have less extreme phenotypes (ADCM, personal observations). It may be that larger stickleback populations in larger lochs facilitate evolution. On South Uist, the eastern lochs are generally smaller, but even the larger ones (e.g., Druidibeg) do not contain unusual stickleback. In any case, the eastern lochs in South Uist, including Druidibeg, have almost neutral pHs, but again it is unclear why. Druidibeg is rather shallow, with a large rocky bottom, and it may be that a shorter residence time of water in the loch (as a consequence of lower volume), coupled with less contact with peat, may prevent the development of more extreme acidity. The topography of South Uist is also rather different from that of the North. North Uist is generally low lying, and catchments drain in a radial pattern, meaning that there is little variation in surface geology within catchments. In contrast, South Uist is hilly in the east and the main catchments drain to the west across the machair. The latter pattern results in a kind of “environmental flow” (movement of water from east to west) that may reduce variation in water chemistry between lochs, at least the development of more alkaline conditions in machair lochs. However, this cannot explain why the eastern lochs are not more acidic. The linear catchments may also facilitate gene flow between lochs on South Uist that is absent on North Uist, and this could inhibit diversification, but the restricted environmental variance alone on South Uist appears sufficient to explain the reduced morphological diversification, without invoking differences in gene flow.

The striking exception provided by stickleback in Loch Trosavat (Tros) to the general pattern of relationship between armor traits and pH is illuminating, if anecdotal. Tros is linked to the sea by a short stream, and this means that large, migratory salmonids (Atlantic salmon, *Salmo salar* and sea trout, *S. trutta*) and potentially other marine fish that prey upon stickleback (e.g., pollock, *P. pollachius*), occur in this loch. This by itself could explain the more developed armor in Tros stickleback. However, this effect is partly driven by the larger size of fish in Trosavat, considering the pH of the water they inhabit. It may be that proximity to the sea results in Tros having a better nutrient status than most acid lochs, which could compound the effect of predation. In the opposite direction, Torm, and especially Scad, have strikingly reduced armor in some individuals missing certain elements such as plates and spines altogether. It is the existence of these fish on North Uist that makes its spectrum of morphological variation especially interesting. While the variation in these lochs is consistent with their low pH, it is possible that additional or subsidiary abiotic or biotic factors are at play, such as unidentified variations in nutritional or ionomic environments.

To conclude, the increased evolutionary variation in North Uist stickleback populations compared to South Uist populations can be explained by greater environmental variation in North Uist, especially linked to variation in the pH of loch water. Research is ongoing to understand the role of pH in the ecology and evolution of Uist stickleback populations, especially nutritional circumstances that may cause constraints on evolution.

## AUTHOR CONTRIBUTIONS

**Mahmuda Begum:** Conceptualization (supporting); data curation (lead); formal analysis (equal); funding acquisition (lead); investigation (lead); methodology (equal); project administration (equal); resources (lead); software (lead); supervision (supporting); validation (equal); visualization (lead); writing – original draft (lead); writing – review and editing (supporting). **Victoria Nolan:** Formal analysis (supporting); software (supporting). **Andrew MacColl:** Conceptualization (lead); data curation (supporting); formal analysis (equal); funding acquisition (supporting); investigation (supporting); methodology (equal); project administration (equal); resources (supporting); software (supporting); supervision (lead); validation (supporting); visualization (supporting); writing – original draft (supporting); writing – review and editing (lead).

## Supporting information


Figure S1

Appendix S1

## Data Availability

Data will be deposited in publicly accessible repository such as Dryad after acceptance of the manuscript.
