# Supplementary figures and images for: Ecological constraint, rather than opportunity, promotes adaptive radiation in three‐spined stickleback (Gasterosteus aculeatus) on North Uist
Source: Ecol Evol. 2023 Jan 10;13(1):e9716. doi: 10.1002/ece3.9716 (PMC9831901; doi:10.1002/ece3.9716)

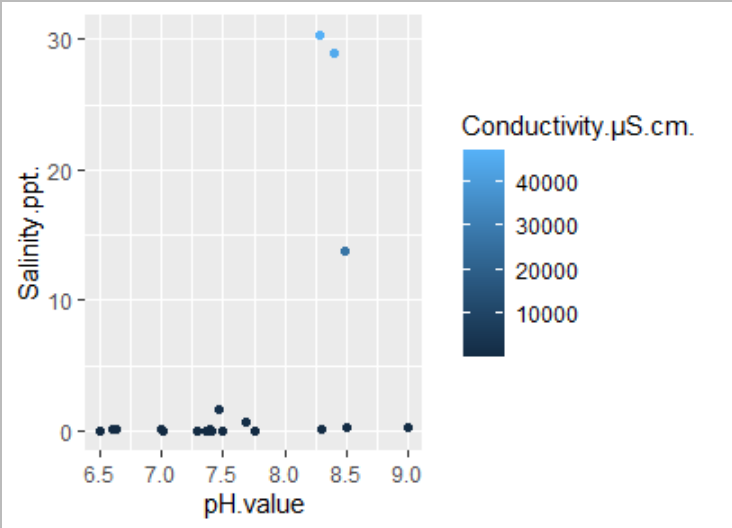


Figure S1. The relationship between salinity and pH for lochs on North and South Uist.

Supplement: Supplementary file 1 — Figure S1 [file ECE3-13-e9716-s002.docx]
