# Supplementary material for: Ecological constraint, rather than opportunity, promotes adaptive radiation in three‐spined stickleback (Gasterosteus aculeatus) on North Uist
Source: Ecol Evol. 2023 Jan 10;13(1):e9716. doi: 10.1002/ece3.9716 (PMC9831901; doi:10.1002/ece3.9716)
Supplement: Supplementary file 2 — Appendix S1 [file ECE3-13-e9716-s001.docx]

**Appendix**

**Table S1. Sampling locations, habitat types, sample number (N) and physicochemical parameters of eight lochs on South Uist, Scotland (FW = Fresh water, SW = Saltwater, Anad = Anadromous, Resi = Resident). Site 8 was an estuary that was sampled at three nearby locations.**

| **Sl.** | **Island** | **Loch** | **Location** | **Habitat** | **N** | **pH** | **Conduc-tivity**  **(µS/cm)** | **Salinity (ppt)** | **Date of sample collection** |
| --- | --- | --- | --- | --- | --- | --- | --- | --- | --- |
| 1. | South Uist | a’Mhoil (Mhoi) | 57°17'11"N;7°25'06"W | FW high pH | 11 FW | 7.5 | 63.9 | 0.04 | 08.05.2019 |
| 2. |  | Grogarry (GroS) | 57°19'59"N; 7°22'55"W | FW high pH | 5 FW | 7.7 | 1167 | 0.746 | 08.05.2019 |
| 3. |  | Eadaray (Eada) | 57°15'39"N; 7°22'14"W | FW high pH | 5 FW | 7.7 | 60.5 | 0.038 | 08.05.2019 |
| 4. |  | West Loch Ollay  (OllW) | 57°15'58"N; 7°24'01"W | FW neutral pH | 30 FW | 7.4 | 138.2 | 0.088 | 08.05.2019 |
| 5. |  | Stilligarry (Stil) | 57°19'09"N; 7°22'12"W | FW neutral pH | 3 FW | 7.4 | 116.5 | 0.074 | 08.05.2019 |
| 6. |  | a' Phuirt-ruaidh  (Phui) | 57°17'48"N; 7°21'54"W | FW neutral pH | 1 Anad  11 Resi | 7.3 | 48.2 | 0.038 | 08.05.2019 |
| 7. |  | Druidibeg (Drui) | 57°19'26"N; 7°19'38"W | FW neutral pH | 10 FW | 7.3 | 40.9 | 0.026 | 08.05.2019 |
| 8. |  | Abhainn Roag  (Aroa 1,2,3) | 57°17'17"N; 7°21'58"W  57°17'27"N; 7°22'33"W  57°17'30"N; 7°22'47"W | FW neutral pH  Brackish water | 13 Anad  38 Resi | 7.4  7.0  7.4 | 52.2  106.8  2630 | 0.033  0.068  1.683 | 06.05.2019 08.05.2019 |

**Table A2. Sampling locations, habitat types, sample number (N) and physicochemical parameters of ten lochs at North Uist, Scotland (FW = Fresh water, SW = Saltwater, Anad = Anadromous, Resi = Resident).**

| **Sl.** | **Island** | **Loch** | **Location** | **Habitat** | **N** | **pH** | **Conduc-tivity**  **(µS/cm)** | **Salinity (ppt)** | **Date of sample collection** |
| --- | --- | --- | --- | --- | --- | --- | --- | --- | --- |
| 1. | North Uist | Chadha Ruaidh (Chru) | 57°35'37"N; 7°11'44"W | FW low pH | 15 FW | 6.6 | 148 | 0.094 | 14.05.2019 |
| 2. |  | Scadavay (Scad) | 57°35′6"N; 7°14′10"W | FW low pH | 12 FW | 6.5 | 130.9 | 0.083 | 15.05.2019 |
| 3. |  | Tormasad (Torm) | 57°33′45"N; 7°19′1"W | FW low pH | 14 FW | 7.0 | 162.5 | 0.104 | 13.05.2019 |
| 4. |  | Trosavat (Tros) | 57°35'3"N; 7°24'45"W | FW low pH | 5 Anad  9 Resi | 6.6 | 165.9 | 0.106 | 14.05.2019 |
| 5. |  | Hosta (Hosta) | 57°37′40"N; 7°29′18"W | FW high pH | 11 FW | 8.5 | 432 | 0.280 | 13.05.2019 |
| 6. |  | Grogary (Grog) | 57°36′54"N; 7°30′40"W | FW high pH | 5 Anad  10 Resi | 8.3 | 340 | 0.220 | 16.05.2019 |
| 7. |  | na Reival (Reiv) | 57°36'39"N; 7°30'50"W | FW high pH | 12 FW | 9.0 | 439 | 0.280 | 14.05.2019 |
| 8. |  | Ard heiskir (Ardh) | 57°34′48"N; 7°24′48"W | SW / Brackish water) | 7 Anad  7 Resi | 8.3 | 47,400 | 30.33 | 14.05.2019 |
| 9. |  | Fairy Knoll (Faik) | 57°38'7"N; 7°12'54"W | SW / Brackish water) | 7 Anad  7 Resi | 8.5 | 45,100 | 28.86 | 16.05.2019 |
| 10. |  | Loch Duin (Duin) | 57°38'35"N; 7°12'40"W | SW / Brackish water) | 7 Anad  7 Resi | 8.4 | 27,460 | 13.73 | 15.05.2019 |

Fig. S1 Measurement of standard length (SL), dorsal spines (1^st^ and 2^nd^), pelvic spine, pelvis (height and length) and lateral plates in a fully plated stained fish. Pelvis length was measured from the reflection in the mirror.
